# PCR-based molecular identification of two intermediate snail hosts of *Schistosoma mansoni* in Cameroon

**DOI:** 10.1186/s13071-020-04033-1

**Published:** 2020-03-30

**Authors:** Mureille Carole Tchami Mbagnia, Tito Trésor Melachio Tanekou, Alvine Christelle Kengne Fokam, Daniel Nguiffo Nguete, Charles Sinclair Wondji, Flobert Njiokou

**Affiliations:** 1 Parasitology and Ecology Laboratory, Department of Animal Biology and Physiology, Faculty of Science, University of Yaoundé I, Yaoundé, Cameroon; 2 Centre for Research in Infectious Diseases, Yaoundé, Cameroon; 3 Department of Biological Sciences, Faculty of Science, University of Bamenda, Bamenda, Cameroon; 4 Research Unit of Applied Biology and Ecology, University of Dschang, Dschang, Cameroon; 5 Department of Vector Biology, Liverpool School of Tropical Medicine, Pembroke Place, Liverpool, L3 5QA UK

**Keywords:** Molecular taxonomy, *Biomphalaria pfeifferi*, *Biomphalaria camerunensis*, *Schistosoma mansoni*, Vector control, Cameroon

## Abstract

**Background:**

Snails of the genus *Biomphalaria* are intermediate hosts of *Schistosoma mansoni*, the causative agent of the human intestinal schistosomiasis. Two *Biomphalaria* species (*Biomphalaria pfeifferi* and *Biomphalaria camerunensis*) are involved in the transmission in Cameroon, where the disease is present nationwide. However, difficulty in the identification of both vectors impedes proper assessment of the epidemiological burden caused by each species. To overcome this issue, we designed a PCR-based molecular diagnostic tool to improve the identification of these species.

**Methods:**

We analyzed the internal transcribed spacer 2 (ITS2) region of *Biomphalaria* ribosomal DNA (rDNA) using polymerase chain reaction amplification (PCR) and restriction fragment length polymorphism (RFLP).

**Results:**

The amplification of the ITS2 region of *Biomphalaria* snails resulted in a 490 bp fragment and produced two profiles for each species after digestion with the restriction enzyme *Hpa* II. The profile 1 (Bc-HpaII-1: 212-bp and 139-bp bands) for *B*. *camerunensis*, was common in all the sampling points; the profile 2 (Bc-HpaII-2: 212-bp and 189-bp bands), was only observed in the Lake Monoun Njindoun sampling site. *Biomphalaria pfeifferi* profile 1 (Bpf-HpaII-1: 211-bp and 128-bp bands) was common in most of *B. pfeifferi* sampling points; the profile 2 (Bpf-HpaII-2: 289-bp and 128-bp bands) was only observed in Mokolo (Far North Cameroon).The second restriction enzyme *Taq*αI, revealed three band profiles, Bc-TaqαI-1 (243-bp, 136-bp and 118-bp bands) and Bc-TaqαI-2 (244-bp, 136-bp and 99-bp) for *B. camerunensis* and Bpf-TaqαI-1 (242-bp, 135-bp and 107-bp bands) for *B. pfeifferi*. Sequencing analysis revealed the occurrence of six haplotypes for *B. camerunensis* and three haplotypes for *B. pfeifferi*. The level of gene flow was low and the *Biomphalaria* populations were not in demographic expansion according to neutrality tests (Tajima’s *D* and Fu’s *Fs*).

**Conclusions:**

The PCR-RFLP technique revealed genetic diversity in *Biomphalaria* snails, and the combination with the morphological method could improve the identification of *B. pfeifferi* and *B. camerunensis* in Cameroon. This could help focus on the infection to evaluate the transmission risk with respect of the different species and to develop efficient and cost-effective control measures.
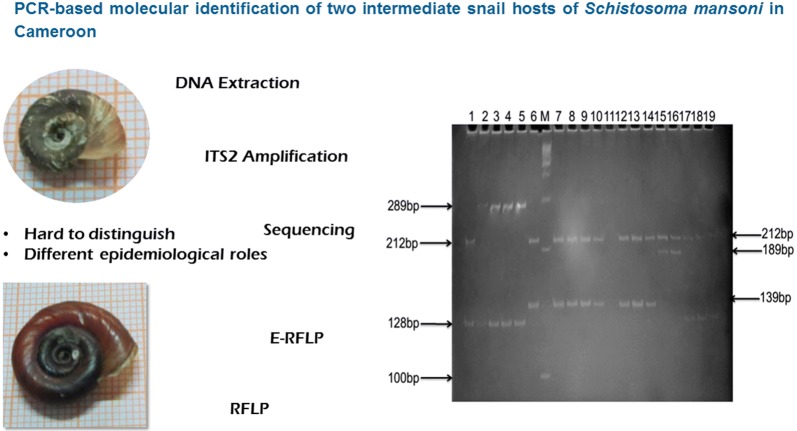

## Background

Schistosomiasis, a chronic disease caused by trematodes of the genus *Schistosoma*, is an important public health problem in many tropical and subtropical areas of the world [1–3]. Indeed, transmission of schistosomiasis is proven in 78 countries, and in 2018 the total number of people in need of preventive chemotherapy was 229.2 million, of which 124.4 million were school-aged children [4].

In Cameroon in 2009, more than 5 million people were at risk of infection with schistosomiasis, and 2 million were known to be currently infected [5]. Intestinal schistosomiasis is the most widely distributed type of schistosomiasis in the equatorial area. This type is caused by the presence in the intestinal vascular system of *Schistosoma mansoni* Sambon, 1907. The Cameroon National Control Programme against schistosomiasis has adopted a periodic large-scale deworming campaign using praziquantel, associated with health education, as control measures. This strategy has considerably reduced morbidity and mortality levels among affected populations. However, most of the old foci remain active, and new foci are observed, likely due to the migration of the infected individuals from endemic areas to schistosomiasis free sites where intermediate hosts are present [6–9]. To overcome this situation and accelerate the progress towards elimination, the Cameroon strategic plan was revised, including the implementation of other control measures such as: (i) access to clean water; (ii) sanitation improvement; and (iii) control of intermediate host populations [10, 11]. This latter control approach requires a better understanding of the distribution, biology and population dynamics of intermediate hosts of schistosomes.

In Cameroon, *S. mansoni* transmission involves two snail species, *Biomphalaria camerunensis* (Boettger, 1941) that has also been recorded in central Africa (from Ghana at the West to the Democratic Republic of Congo at the East) [11, 12] and *Biomphalaria pfeifferi* (Krauss, 1848) that is also present throughout sub-Saharan Africa and also in one country in North Africa (Algeria) [12]. *Biomphalaria pfeifferi* is widely distributed throughout the country and is known as the main intermediate host of *S. mansoni*. Conversely, *B. camerunensis* has until now only been reported under the 6° north latitude and appears to play a minor role in the transmission of *S. mansoni* [13]. However, recent compatibility studies highlighted a relatively high susceptibility and cercarial emission rate in some *B. camerunensis* populations as high as those observed among *B. pfeifferi*, suggesting that they might play a more important role in the transmission of *S. mansoni* [14].

*Biomphalaria pfeifferi* and *B. camerunensis* are very similar morphologically, which makes their routine identification difficult. The last study on the distribution of these snail species in Cameroon was performed nearly 30 years ago and the results are not reflective to the current situation, as there have been significant ecological and human changes in the respective foci. A clear distinction of these *Biomphalaria* spp. snails might help sharpen disease mapping and evaluate the transmission risk in localities where they are found. For many decades, the identification of snail intermediate hosts had been mainly based on the comparison of morphological and morphometric characters of shells [15–17]. Although the shell morphology remains an important taxonomic instrument [18, 19], studies employing alternative tools such as molecular identification seem to be more accurate for closely related species [20–25]. In the last two decades, molecular tools have been evaluated for both snail identification and phylogenetic studies [26–29]; the polymerase chain reaction and restriction fragment length polymorphism (PCR-RFLP) analysis of the internal transcribed spacer (ITS) region of rDNA and the analysis of cytochrome *c* oxidase subunit 1 region of mitochondrial DNA have proven to be cheaper and powerful for the identification of several *Biomphalaria* species [30–35]. More recently, the PCR-RFLP technique has been used to distinguish *Biomphalaria* species in Brazil and it appears to be an alternative molecular tool to their morphological identification [36].

The aim of this study was to test the efficiency of the PCR-RFLP protocol developed for identification of *Biomphalaria* species in South America [34, 36], for the separation of the two main *Biomphalaria* spp. currently found in Cameroon. Furthermore, sequencing of rDNA fragments enabled us to assess the genetic diversity and population structure of both species in Cameroon.

## Methods

### Snail sampling sites and collection method

**Fig. 1:**
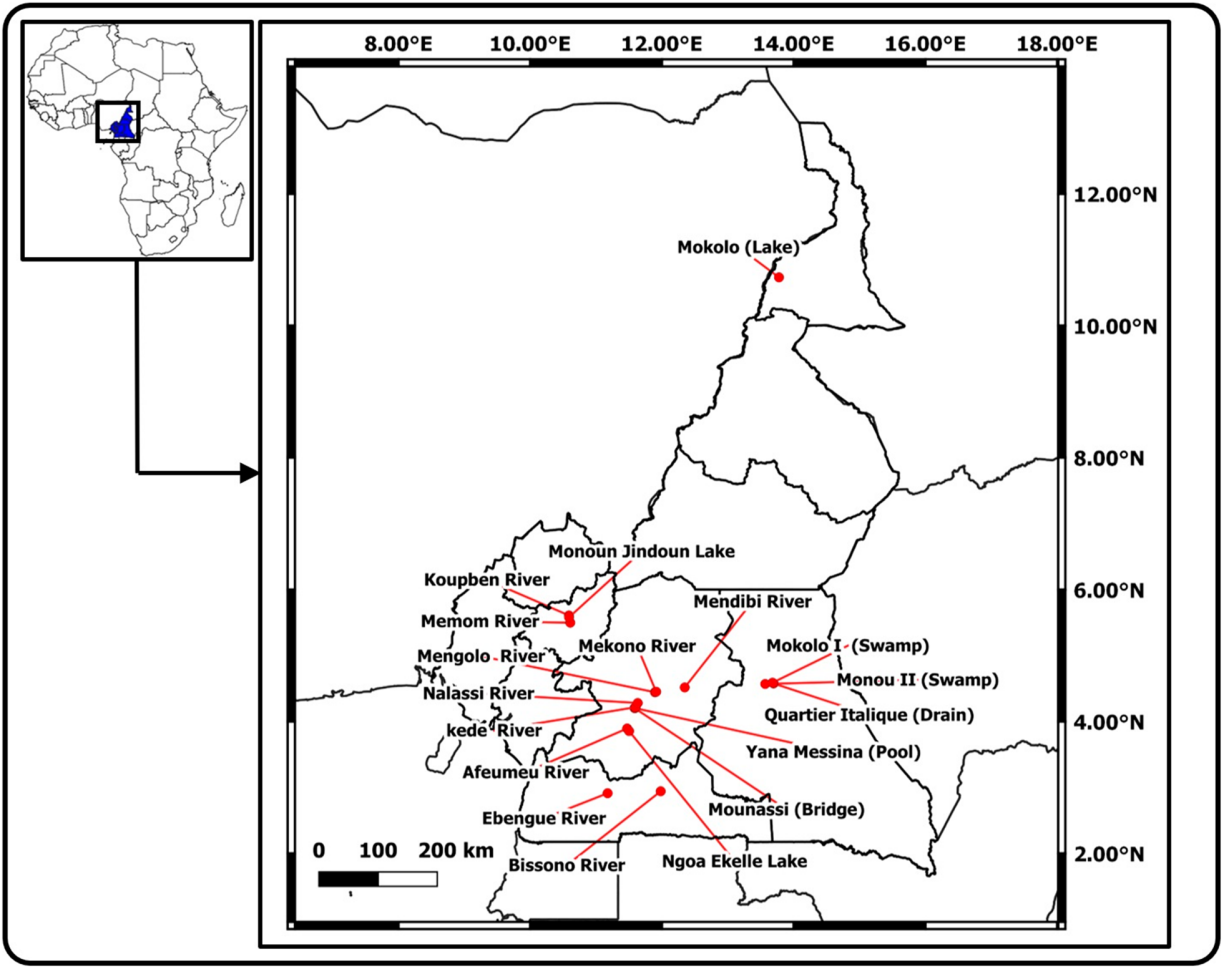
Map showing the different collection sites. Red dots indicate collection sites

Snail sampling was conducted from July to August 2017 in eighteen sites all located in five administrative Regions of Cameroon: nine sites in the Centre, two in the South, three in the West, two in the East and one in the Far North (Table 1, Fig. 1). Sites mentioned in previous studies were visited [13, 14], while new sites were chosen in additional streams favourable to the survival of molluscs. Snails were collected using a long-handled dip net by systematically combing the aquatic vegetation and identified using morphological criteria previously described by Brown [37]. For each sample, some individuals were taken at random and kept alive for further parasites screening, while others were individually fixed in labelled tubes containing 95% ethanol and transferred to the Parasitology and Ecology Laboratory of the University of Yaoundé I, Cameroon, where they were stored at − 20 °C until processing for molecular studies.

**Table 1:** Description of different sampling sites

Region	Bioclimatic characteristics	Town	Water collection (*n*)	Geographical coordinates
Centre	Degraded forest, subequatorial climate	Minkama	Mounassi pond (32 *Bc*)	4°12ʹ14″N, 11°35ʹ0.2″E
			Yana Messina pool (6 *Bc*)	4°12ʹ1″N, 11°35ʹ3″E
			Kede River (32 *Bc*)	4°12ʹ6″N, 11°35ʹ6″E
		Nalassi	Nalassi River (50 *Bc*)	4°16ʹ49″N, 11°37ʹ58″E
		Nkoteng	Mendibi River (6 *Bc*)	4°31ʹ00″N, 12°08ʹ00″E
		Mbandjock	Mengolo River (30 *Bc*)	4°26ʹ37″N, 11°53ʹ40″E
			Mekono River (34 *Bc*)	4°27ʹ04″N, 11°54ʹ46″E
		Yaoundé	Afeme River (30 *Bp*)	3°53ʹ38″N, 11°28ʹ20″E
			Ngoa-Ekellé Lake (30 *Bp*)	3°51ʹ15″N, 11°30ʹ07″E
South	Dense rain forest, Guinean subequatorial climate	Sangmelima	Bissono River (30 *Bc*)	2°56ʹ28″N, 11°58ʹ55″E
		Ebolowa	Ebengue River (4 *Bc*)	2°54ʹ42″N, 11°10ʹ33″E
East	Equatorial forest, subtropical climate	Bertoua	Monou II swamp (30 *Bc*)	4°34ʹ14″N, 13°41ʹ34″E
			Quartier Italie drain (30 *Bc*)	4°34ʹ35″N, 13°41ʹ43″E
			Mokolo I swamp (30 *Bc*)	4°35ʹ18″N, 13°40ʹ48″E
West	Shrub savannah, equatorial climate	Kouoptamo	Koupben River (30 *Bc*)	5°36ʹ33″N, 10°35ʹ17″E
		Mangoum	Memom River (30 *Bc*)	5°29ʹ57″N, 10°36ʹ30″E
		Monoun Njindoun	Monoun Njindoun Lake (32 *Bc*)	5°34ʹ53″N, 10°35ʹ23″E
Far North	Savannah, tropical Sudan-Sahel climate	Mokolo	Lake (4 *Bp*)	10°44ʹ00″N, 13°46ʹ4″E

*Abbreviations*: n, number of snails collected; *Bc*, *Biomphalaria camerunensis*; *Bp*, *Biomphalaria pfeifferi*

### DNA extraction

Total DNA was extracted from the foot of 470 *Biomphalaria* snails (around thirty individuals per sampling site) using a cethyl trimethyl ammonium bromide (CTAB)-based protocol as described by Maniatis et al. [38]. Samples were thawed and air-dried; briefly, each snail foot was homogenized with a pestle in 600 µl of CTAB buffer (CTAB 2%; 0.1 M Tris-HCl, pH 8; 0.02 M EDTA, pH 8; 1.4 M NaCl). The homogenized mixture was incubated at 60 °C for 30 min. After this incubation, the DNA was extracted using 600 µl of chloroform/isoamylic alcohol (24/1; V/V) mixture, and then precipitated with isopropanol (V/V). After a centrifugation at 10,000×*g* for 15 min, the DNA pellet was washed with 70% ethanol, air-dried, and re-suspended in sterile water. DNA samples were stored at − 20 °C until PCR amplification.

### PCR amplification of the ITS2 region of *Biomphalaria* spp

The ITS2 region of *Biomphalaria* spp. was amplified by PCR, using the primers ITS2F (5ʹ-CGT CCG TCT GAG GGT CGG TTT GC-3ʹ) [30] and ETTS1 (5ʹ-TGC TTA AGT TCA GCG GGT-3ʹ) [39] hybridizing in the conserved areas of the *5.8S* and *28S* ribosomal genes, respectively. The PCR amplification was undertaken in a final reaction volume of 20 μl containing 2 µl of extracted DNA, 2 µl of TBE PCR buffer (10×), 0.8 μl of each primer (10 µM), 0.4 μl of dNTPs mixture (10 mM), 0.06 μl of *Taq* DNA polymerase (5 U/µl) and 13.94 μl of sterile water. DNA was amplified in a Techne TC-412 (Bibby Scientific Limited, Staffordshire, UK) thermal cycler under the following cycling conditions: initial denaturation at 95 °C for 3 min 30 s, followed by 35 cycles each at 95 °C for 30 s, 60 °C for 30 s and 72 °C for 30 s, and a final extension step at 72 °C for 10 min. The amplicons were resolved on 2% agarose gels stained with ethidium bromide and visualized under UV light.

### Purification, sequencing and *in silico* RFLP

Purification of some amplified DNA samples was made using the enzymatic PCR clean up method with Exonuclease I (*Exo*I) and shrimp alkaline phosphatase (SAP) (New England Biolabs, Boston, MA, USA) which offer an easy way to remove the remaining primers and dNTPs left from the PCR reaction. The DNA concentration was then measured using a NanoDrop™ spectrophotometer (Thermo Fisher Scientific, Wilmington, USA) and samples complying with the minimal concentration (ng/µl) were directly sequenced commercially (GENEWIZ, Liverpool, UK). The sequences obtained were visualized and edited using BioEdit software, then confirmed *via* alignment to similar sequences using the nucleotide BLAST tool on GenBank. Once confirmed, the putative *B. pfeifferi* and *B. camerunensis* sequences were subjected to *in silico* RFLP using the Restriction Mapper online tool (http://www.restrictionmapper.org/) to choose restriction enzymes which could clearly distinguish the two *Biomphalaria* species.

### Digestion and revelation of profiles

The two enzymes which exhibited good digestion profiles after *in silico* RFLP (*Hpa*II and *Taq*αI) were used for the digestion of the PCR products. The reaction was carried out in a final volume of 25 µl, containing 2 μl of amplified DNA, 1 μl of restriction enzyme, 5 μl of the manufacturerʼs buffer and 17 μl of sterile water, at 37 °C for 2 h. After digestion, the products were resolved on 8% polyacrylamide gel stained with ethidium bromide and visualized under UV light.

### Phylogenetic and population structure analyses

All the sequences were aligned in BioEdit with the ClustalW [40] algorithm for phylogenetic analyses. The evolutionary history was inferred using the Maximum Likelihood method based on the Jukes-Cantor model [41]. Initial trees for the heuristic search were obtained automatically by applying Neighbor-Join and BioNJ algorithms to a matrix of pairwise distances estimated using the Maximum Composite Likelihood approach, and then selecting the topology with highest log likelihood value. Bootstrap values supporting the nodes were computed as the percentage of trees in which associated taxa clustered together. Evolutionary analyses were conducted in MEGA7 [42].

Estimation of genetic diversity, including polymorphic sites (S), haplotype diversity (Hd), nucleotide diversity (π), were performed using DnaSP5.10.01 [43]. Haplotype diversity was considered as the probability that two randomly sampled alleles are different, and nucleotide diversity was considered as the average number of nucleotide differences per site in pairwise comparisons among DNA sequences [44]. Genetic differentiation among populations (*F*_*ST*_), Fu’s *Fs* statistics and Tajima’s *D* [45] values were also estimated using the software DnaSP 5.10.01 [43]. To better visualize the phylogenetic relationships among haplotypes, a haplotype network was constructed among the defined haplotypes using TCS v1. 21 software [46] and mutations steps were generated using DnaSP 5.10.01 [43] and MEGA7 [41].

## Results

### ITS2 amplification and sequencing

The PCR amplification of *Biomphalaria* ITS2 region resulted in a product of 490 bp. After sequencing, editing and aligning to a reference sequence, four amplicon sizes were obtained: 478 bp and 485 bp for *B. camerunensis*; 486 bp and 497 bp for *B. pfeifferi*.

### Digestion and revelation of profiles

The digestion of PCR products using restriction enzymes (*Hpa*II and *Taq*αI) revealed different profiles for the two *Biomphalaria* species.

**Fig. 2:**
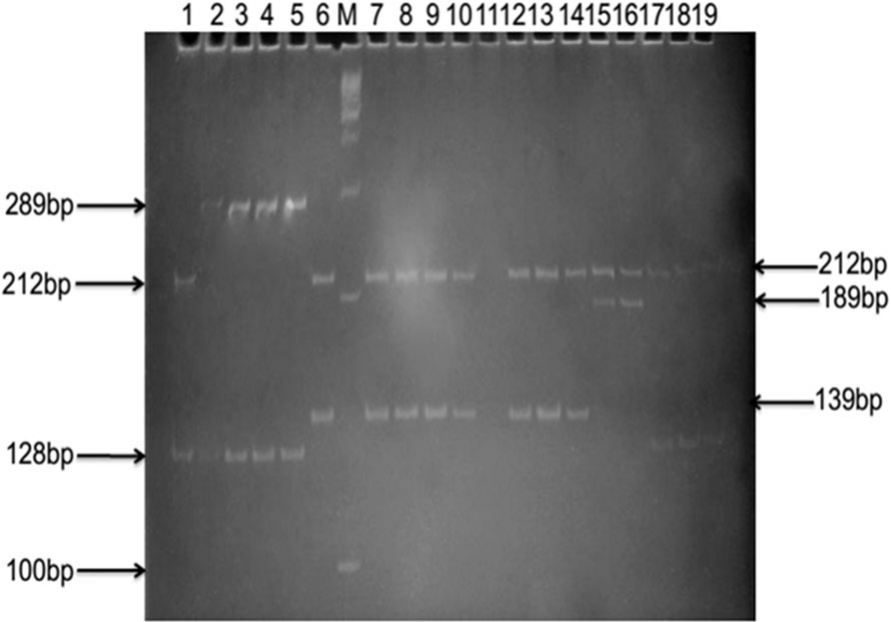
Polyacrylamide gel showing RFLP profiles obtained after the digestion with *Hpa*II of *Biomphalaria* ITS2. Lane 1: *B. pfeifferi* profile 1; Lanes 2–5: *B. pfeifferi* profile 2; Lane 6: *B. camerunensis* profile 1; Lane M: molecular weight marker (100 bp); Lanes 7–10: *B. camerunensis* profile 1; Lane 11: no sample; Lanes 12–14: *B. camerunensis* profile 1; Lanes 15, 16: *B. camerunensis* profile 2; Lanes 17–19: *B. pfeifferi* profile 1

*Biomphalaria camerunensis* individuals exhibited two profiles, each with two bands: profile 1 (Bc-HpaII-1: 212-bp and 139-bp bands) was common in all the sampling points; profile 2 (Bc-HpaII-2: 212-bp and 189-bp bands) was only observed in the Lake Monoun Njindoun sampling site in the West Region and was displayed by ~ 90% of the individuals sampled there. Individuals of *B. pfeifferi*, also presented two profiles, each with two bands: profile 1 (Bpf-HpaII-1: 211-bp and 128-bp bands), was common in all *B. pfeifferi* sampling points; whereas profile 2 (Bpf-HpaII-2: 289-bp and 128-bp bands) was only observed in Mokolo (Far North Cameroon) and was present in 95% of the sampled individuals (Fig. 2).

**Fig. 3:**
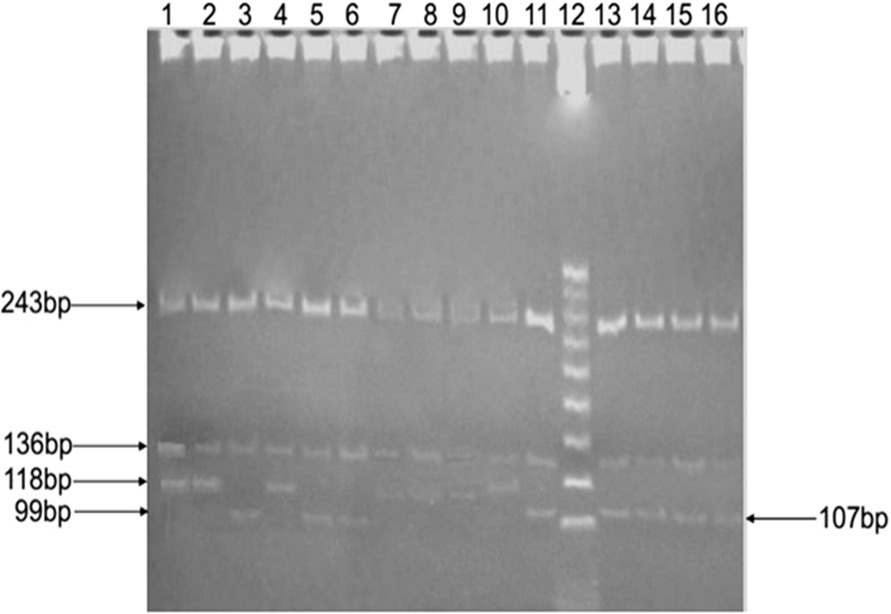
Polyacrylamide gel showing RFLP profiles obtained after the digestion with *Taq*αI of *Biomphalaria* ITS2. Lanes 1, 2: *B. camerunensis* profile1; Lane 3: *B. camerunensis* profile 2; Lane 4: *B. camerunensis* profile1; Lanes 5, 6: *B. camerunensis* profile 2; Lanes 7–10: *B. camerunensis* profile1; Lane 11: *B. Pfeifferi*; Lane 12: molecular weight marker (25 bp); Lanes 13–16: *B. pfeifferi*

Figure 3 shows the different profiles obtained for the two *Biomphalaria* species after the digestion of ITS2 amplicons with *Taq*αI enzyme. Individuals of the species *B. camerunensis* showed two profiles, each represented on the gel by three bands: profile 1 (Bc-TaqαI-1: 243-bp, 136-bp and 118-bp bands) was common in all the sampling sites; while profile 2 (Bc-TaqαI-2: 244-bp, 136-bp and 99-bp bands) was only observed in samples from Lake Monoun Njindoun (these were the same individuals that displayed profile 2 with the *Hpa*II enzyme). All the individuals of *B. pfeifferi* presented a three-band profile (242 bp, 135 bp and 107 bp).

### Phylogenetic analyses

**Fig. 4:**
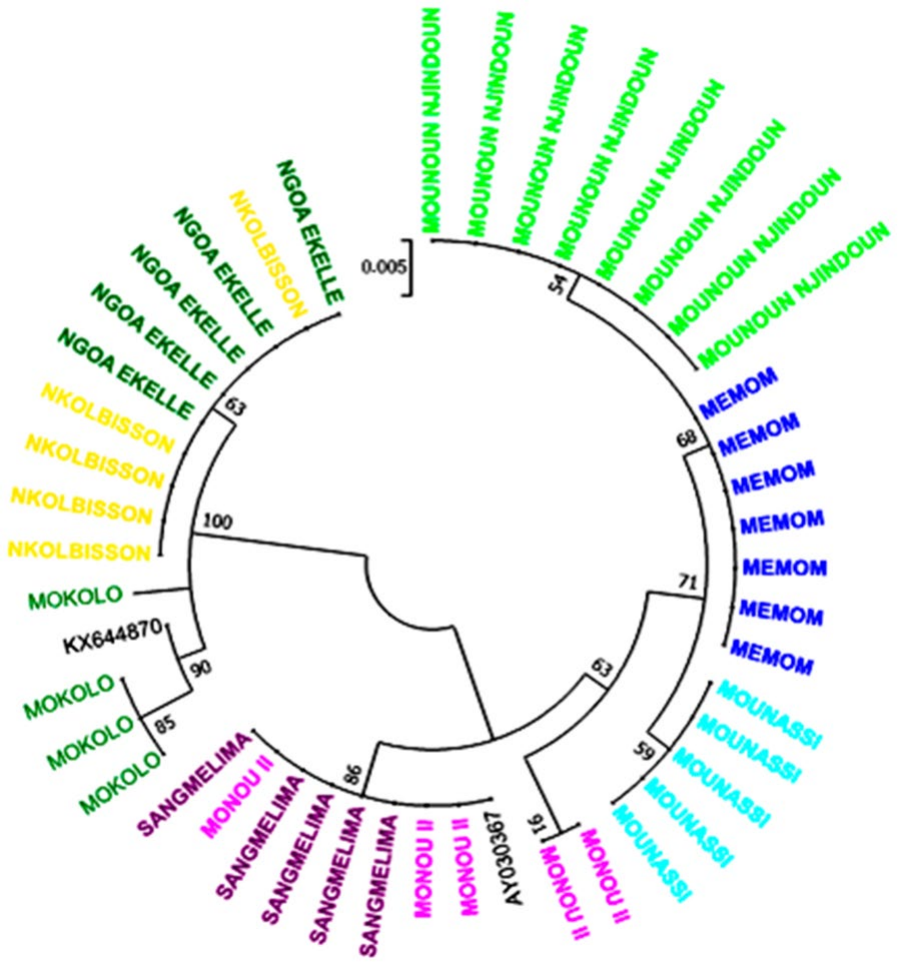
Maximum likelihood phylogenetic tree constructed with ITS2 sequences. AY030367: reference ITS accession number of *B. camerunensis*; KX644870: reference ITS accession number of *B. pfeifferi*

**Fig. 5:**
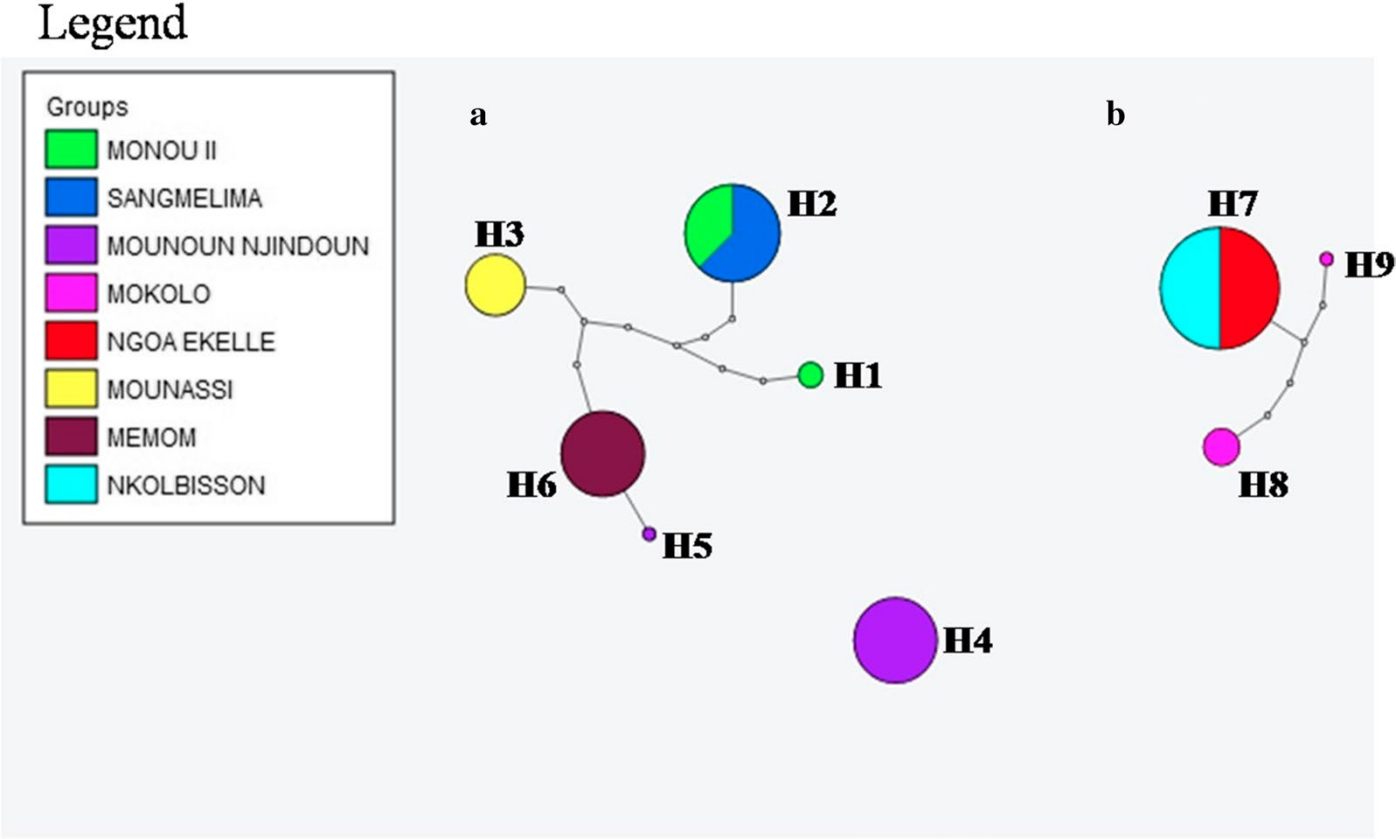
Haplotype networks for *B. camerunensis* (**a**) and *B. pfeifferi* (**b**). H1-H9: different haplotypes; the sizes of the circles are proportional to the haplotype frequencies

**Fig. 6:**
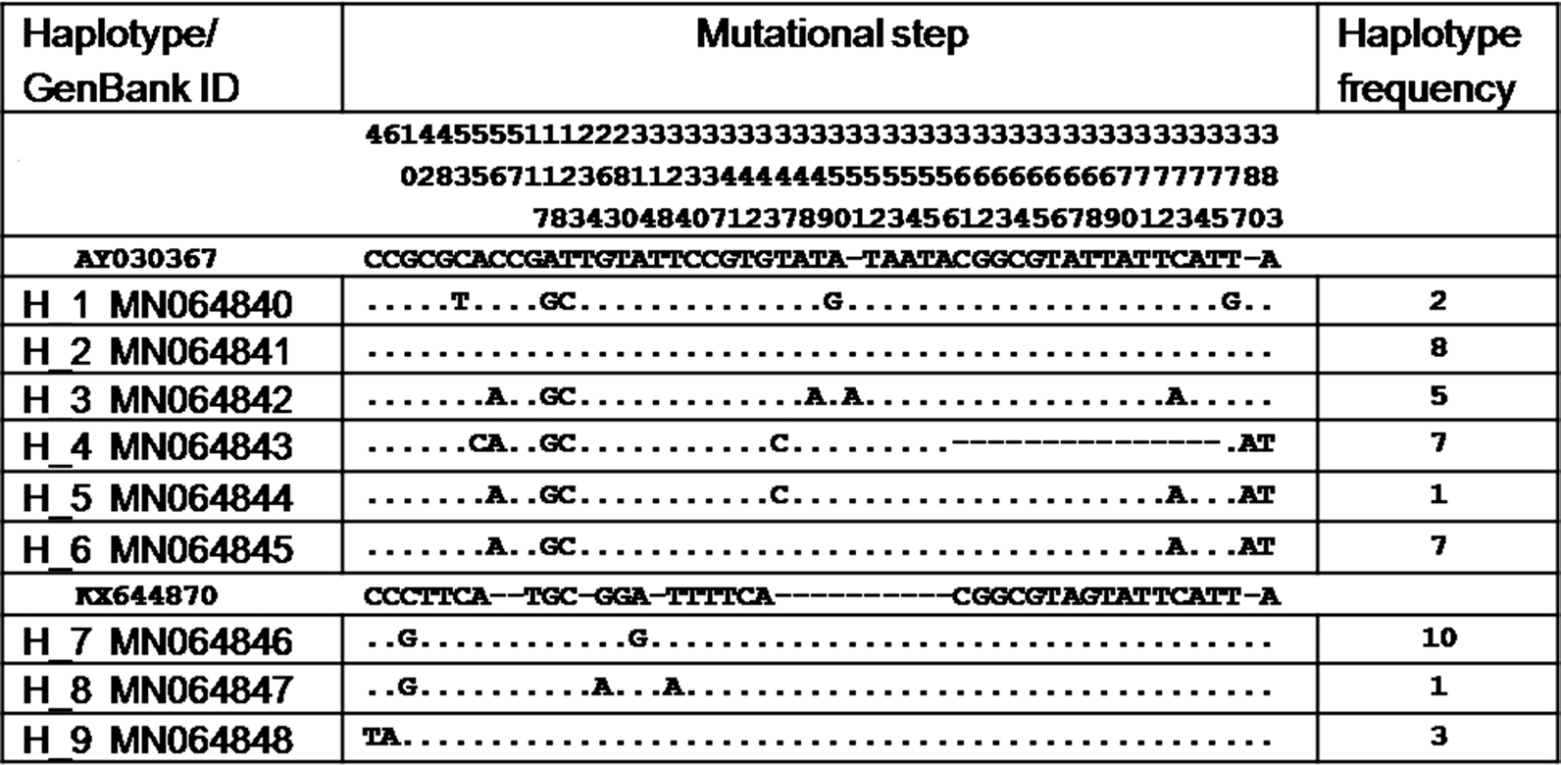
Different mutational steps of haplotypes obtained and accession numbers of their sequences on GenBank. AY030367, reference ITS accession number of *B. camerunensis*; KX644870, reference ITS accession number of *B. pfeifferi*

The analysis involved 44 nucleotide sequences of 424 bp plus two reference nucleotide sequences, one for *B. pfeifferi* from the South-East of Nigeria [47] and one for *B. camerunensis* from Sangmelima in Cameroon [48]. A clear segregation of the two *Biomphalaria* species (bootstrap 100) was observed. One cluster included *B. pfeifferi* from Mokolo in the Far North Region, Nkolbisson and Ngoa Ekelle in the Centre Region, and another cluster included *B. camerunensis* from Mounassi in the Centre Region, Sangmelima in the South Region, Monou II in the East Region, Monoun Njindoun and Memom in the West Region (Fig. 4). A total of nine haplotypes were detected in the different studied populations including six for *B. camerunensis* and three for *B. pfeifferi*. In general, haplotypes of each species were strongly related with a maximum of 5 and 8 mutational steps in *B. pfeifferi* and *B. camerunensis* populations, respectively (Fig. 5). The haplotype H4 found only in Monoun Njindoun in the West Region was the only haplotype isolated from the other haplotypes of *B. camerunensis* and was different from the reference sequence from Cameroon with 22 mutational steps (Fig. 6). When comparing the haplotypes of the two species, 30 mutational steps were recorded, confirming the segregation of these species (Fig. 6).

### Genetic diversity of *Biomphalaria* populations

Of the eight populations analysed, six did not display any polymorphism (all the individual having the same haplotype). Haplotype and nucleotide diversities of *B. camerunensis* samples were 0.798 and 0.008, respectively, while in *B. pfeifferi* samples, they were 0.473 and 0.004, respectively. Assessment of demographic expansion of populations, based on neutrality tests gave a negative value for Tajima’s *D* in Mokolo (− 0.796) and a positive value (1.718) in Monou II and these values were not significant; Fuʼs *Fs* test values were positive in samples of the two species (Table 2), showing that populations under study were not in expansion.

**Table 2:** Haplotype diversity and nucleotide diversity among populations of *Biomphalaria camerunensis* and *Biomphalaria pfeifferi*

Population	*n*	H	S	Hd	Π	*D*	Fu’s *Fs*
*Biomphalaria camerunensis*							
Monou II	5	2	6	0.600	0.008	1.718	3.967
Sangmelima	5	1	0	0.000	0.000	–	–
Mounassi	5	1	0	0.000	0.000	–	–
Monoun Njindoun	8	1	0	0.000	0.000	–	–
Memom	7	1	0	0.000	0.000	–	–
Overall	30	5	10	0.798	0.008	0.934	3.190
*Biomphalaria pfeifferi*							
Nkolbisson	5	1	0	0.000	0.000	–	–
Ngoa-Ekellé	5	1	0	0.000	0.000	–	–
Mokolo	4	2	5	0.500	0.006	-0.796	2.59
Overall	14	3	6	0.473	0.004	-0.139	2.301

*Abbreviations*: *n*, population sample size; H, number of haplotypes; S, number of substitutions; Hd, haplotype diversity; π, nucleotide diversity; *D*, Tajima’s index; –, no polymorphism

### Population structure

Genetic structure of *Biomphalaria* spp. populations was analyzed by pairwise *F*_*ST*_ among five populations of *B. camerunensis* and three populations of *B. pfeifferi*. *F*_*ST*_ values were high and significant (*P* < 0.01) between all *B. camerunensis* pairs from Sangmelima, Monou II, Mounoun Njindoun, and Memom but low and non-significant between the two populations from Sangmelima in the South Region and Monou II in the East Region (*P* = 0.25) (Table 3). In *B. pfeifferi* populations, *F*_*ST*_ values were only significant between the samples from the Center Region (Nkolbisson, Ngoa-Ekelle) and the one from the Far North Region (Mokolo) (*P* < 10^−3^) (Table 4).

**Table 3:** Fixation index (*F*_*ST*_) between *Biomphalaria camerunensis* populations

Locality	Mounassi	Monoun Njindoun	Memom	Monou II	Sangmelima
Mounassi (*n* = 5)	–				
Monoun Njindoun (*n* = 8)	1**	–			
Memom (*n* = 7)	1**	1***	–		
Monou II (*n* = 5)	0.7**	0.7**	0.7**	–	
Sangmelima (*n* = 5)	1**	1***	1**	0.25	–

*Notes*: Numbers in parentheses indicate the number of individuals analyzed

**P < 0.01; ***P < 0.0001

**Table 4:** Fixation index (*F*_*ST*_) between localities *Biomphalaria pfeifferi* populations

Localities	Ngoa-Ekellé	Nkolbisson	Mokolo
Ngoa-Ekellé (*n* = 5)			
Nkolbisson (*n* = 5)	0		
Mokolo (*n* = 4)	0.6**	0.6**	-

*Note*: Numbers in parentheses indicate the number of individuals analyzed

**P < 0.01

## Discussion

Morphological identification of *S. mansoni* vector species of the genus *Biomphalaria* is difficult because shells are very similar in shape and color. Moreover, morphological and anatomical characters were found to vary among populations as shown in Sangmelima and Nkoteng by Peka Sangou (2010, personal communication) and Mvogo Ndongo (2012, personal communication). To scale up the fight against schistosomiasis and achieve elimination, an anti-vector campaign has recently been advocated in addition to MDA which for a very long time had been the only prevention approach [49]. The identification of the two sibling *Biomphalaria* spp. is tricky, but useful for the establishment of baseline data, prior to a vector control campaign. To provide more tools to achieve this goal, we designed a diagnostic assay that will facilitate the morphological identification of *B. pfeifferi* and *B*. *camerunensis* in Cameroon and help improve the control of these species.

### Taxonomic study

The PCR-RFLP-based protocol used has clearly distinguished *B. camerunensis* species (profiles Bc-HpaII-1 and Bc-HpaII-2) from *B. pfeifferi* species (profiles Bpf-HpaII-1 and Bpf-HpaII-2) as was the case for Brazilian *Biomphalaria* species [35]. The same technique had already proven useful in the identification of other species such as species of the *Anopheles gambiae* complex [50]. This technique is a promising simple tool which can help to overcome the misidentification of sibling vectors and thus help to understand epidemiological issues. Moreover, the PCR-RFLP technique revealed an intraspecific variation, characterized by the occurrence of two ribotypes in each *Biomphalaria* species. As no intermediate profile was found between ribotypes, further investigation is needed to check their taxonomical status (subspecies or simple variant). The existence of intraspecific variation is in line with the observation of a high polymorphism in the susceptibility of *B. camerunensis* populations to *S. mansoni* [14]. This level of polymorphism is expected in an outbred species such as *B. camerunensis* [51].

The phylogenetic tree constructed with ITS2 sequences clearly segregated the individuals of the two species, thus confirming their divergence; however, within each species, the two main related branches of the tree correspond to the two different profiles obtained and are indicative of an intraspecific variation. Molecular phylogenies already helped in the classification of several mollusc taxa like bivalves [52], it is then an added value for taxonomical studies.

### Molecular diversity and population dynamics

Phylogenetic studies showed that almost all the haplotypes obtained within each species, regardless of their geographical origin, were closely related. Among the five populations of *B*. *camerunensis*, Monou II showed the highest haplotype diversity (Hd: 0.600) and a low nucleotide diversity (π: 0.008), which are indicative of a rapid population growth from a small-sized ancestral population in which the time has not been enough to find a strong diversity between haplotypes [53]. The low values of Hd (0.473) and π (0.004) obtained in *B. pfeifferi* populations are indicative of a severe and prolonged bottleneck; however, more investigations are necessary to confirm these suggestions. In addition, pairwise *F*_*ST*_ between the samples from the different administrative regions studied were high and significant, showing no signal of gene flow between these populations. Nevertheless, the lack of polymorphism in the genetic marker used could lead to a less accurate estimation of this parameter. The low genetic diversity observed in the studied populations can result from their mating system or from their ecology, since low genetic diversity is severe in subdivided populations with seasonal variation in abundance [1]. Effective population sizes and density variation appear to be important factors in the loss of variability and *Biomphalaria* are known to have subdivided habitats [54]. In addition, the positive values of the Tajima’s *D* and Fu’s *Fs* neutrality tests show that all the populations studied are not currently expanding. This information shows that vector control performed now will lead to a rapid population density decrease in *Biomphalaria* spp., but other studies using other nuclear or mitochondrial polymorphic markers are needed to provide more accurate demographic parameters.

## Conclusions

The aim of this study was to provide a simple tool that can help to easily and accurately identify *B. pfeifferi* and *B. camerunensis* from Cameroon and provide some initial data on their genetic diversity. The digestion of ITS2 DNA fragments with *Hpa*II and *Taq*αI allowed to clearly distinguish the two *Biomphalaria* species present in Cameroon, showing that the protocol used constitutes a useful alternative that can help to accurately identify the two species and thus help in future vector control campaigns. The genetic variability observed within *B. camerunensis* is in line with the ability of some of its populations to transmit schistosomes at the same level as the former known principal host, *B. pfeifferi*. Further investigations are required to formally confirm the link between the vector competence of some *B. camerunensis* populations and their genetic background.

## Data Availability

Data supporting the conclusions of the article are included within the article. The newly generated sequences were submitted to the GenBank database under the accession numbers MN064840-MN064845 (*Biomphalaria camerunensis*) and MN064846-MN064848 (*Biomphalaria pfeifferi*). The datasets used and/or analyzed during the current study are available from the corresponding author upon reasonable request.

## Abbreviations

MDA: Mass drug administration
DNA: Deoxyribonucleic acid
ITS: Internal transcribed spacer
PCR: Polymerase chain reaction
RFLP: Restriction fragment length polymorphism
